# Bibliographical

**Published:** 1878-10

**Authors:** 


### BIBLIOGRAPHICAL.

Robert Clarke & Co., of this city, have just issued "A Catalogue of American and British Books," embracing periodicals, transactions, etc., relating to medicine, surgery, dentistry, pharmacy, chemistry and kindred subjects, classified by subjects, with an index by authors.

In the preparation of this catalogue it was the aim to present in a new form a convenient hand book which may be of service to those selecting books for purchase.

It embraces two thousand, three hundred of the principal American and British Books on medical and allied topics, which have been properly classified and arranged for ready reference. Such works only as are in print and readily obtainable are included in this catalogue.

This is an invaluable work to those who wish to supply themselves with the best works of the various branches of medical science. It will be sent by mail to any who may wish it. Price twenty-five cents.

